# Antiviral Activities of Oleanolic Acid and Its Analogues

**DOI:** 10.3390/molecules23092300

**Published:** 2018-09-09

**Authors:** Vuyolwethu Khwaza, Opeoluwa O. Oyedeji, Blessing A. Aderibigbe

**Affiliations:** Department of Chemistry, University of Fort Hare, Alice Campus, Alice 5700, Eastern Cape, South Africa; vuyolwethukhwaza@gmail.com (V.K.); ooyedeji@ufh.ac.za (O.O.O)

**Keywords:** HIV, influenza virus, HBV/HCV, natural product, triterpenoids, medicinal plant

## Abstract

Viral diseases, such as human immune deficiency virus (HIV), influenza, hepatitis, and herpes, are the leading causes of human death in the world. The shortage of effective vaccines or therapeutics for the prevention and treatment of the numerous viral infections, and the great increase in the number of new drug-resistant viruses, indicate that there is a great need for the development of novel and potent antiviral drugs. Natural products are one of the most valuable sources for drug discovery. Most natural triterpenoids, such as oleanolic acid (OA), possess notable antiviral activity. Therefore, it is important to validate how plant isolates, such as OA and its analogues, can improve and produce potent drugs for the treatment of viral disease. This article reports a review of the analogues of oleanolic acid and their selected pathogenic antiviral activities, which include HIV, the influenza virus, hepatitis B and C viruses, and herpes viruses.

## 1. Introduction

Viral diseases remain a major problem for humankind. It has been reported in some reviews that there is an increase in the number of viral diseases responsible for death and morbidity around the world [1,2]. According to the recent release from the National Health Laboratory Service (NHLS), influenza kills approximately 6000–11,000 South Africans every year [3]. Although other treatments eradicate some pathogens, such as polio, mumps and smallpox, other viral diseases, such as the hepatitis C virus (HCV) and the human immunodeficiency virus (HIV), have proven difficult to combat using the conventional treatment approach. In addition, the increase in viral resistance to drugs, as well as the serious adverse effects of antiviral drugs, results in serious medical problems, particularly when drugs are administered in combination over a prolonged treatment period [4]. Thus, there is a great need to develop novel potential antiviral agents from different sources, such as medicinal plants or natural products, which have been used in many regions as antiviral agents for many years [5,6,7].

**Figure 1:**
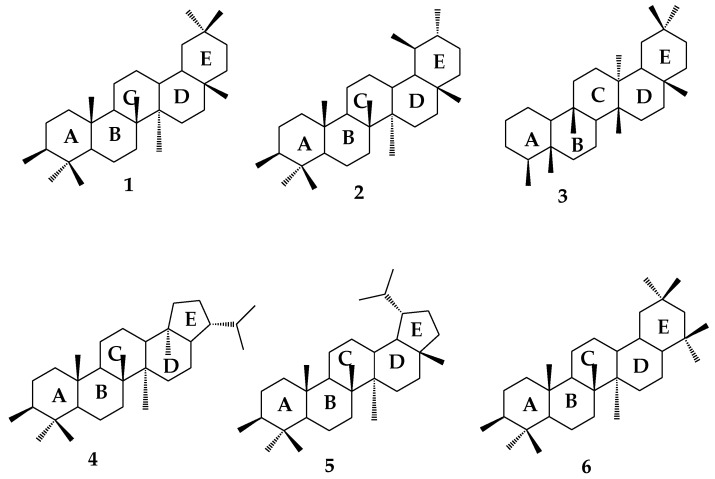
Types of pentacyclic triterpenes structures.

Natural products have been proven as the main source of biologically active compounds, and they are potentially useful for drug development [8]. Pentacyclic triterpenes (PTs) are the most significant group of phytochemicals synthesized from plants through cyclization of squalene, and are known as a large class of secondary plant metabolites that are constructed by isoprene (2-methylbutadiene) (C5H8) units [9]. Structurally, they contain 5- and 6-membered rings (A, B, C, D, and E). The carbon skeleton of PTs is divided into six different subgroups: Oleanane (**1**), ursane (**2**), friedelane (**3**), hopane (**4**), lupine (**5**), and gammacerane (**6**) (Figure 1) [10]. It has been reported that approximately 20,000 triterpenoids exist in nature [11,12].

**Figure 2:**
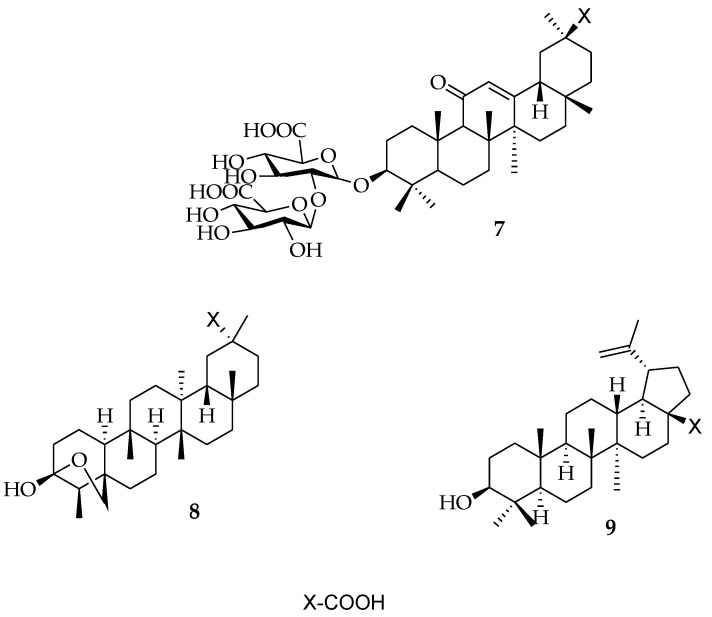
Isolated antiviral compounds from plants.

Antivirals are compounds which prevent viral development. Some of these antivirals can be isolated from sources, such as plants, animals, bacteria or fungi, while others can be obtained by chemical synthesis [13,14]. Antiviral mechanisms identified from natural products have shed light on how they interact with the viral life cycle, such as viral entry, replication, assembly, and release, as well as targeting of virus–host-specific interactions [5,6]. The antiviral properties of PTs have attracted the attention of many researchers. Pompei and colleagues extracted glycyrrhizic acid (Figure 2) (**7**) from the crude extract of *Glycyrrha glabra* roots, and it was found to be active against the herpes simplex virus [15]. Chen et al., also isolated an oleanane-type triterpene derivative known as salaspermic acid (Figure 2) (**8**) from *Tripterygium wilfordii*, which blocked the replication of HIV in H9 lymphocytes (IC_50_: 10 μM) [16]. Toshihiro et al. also isolated betulinic acid (BA) (Figure 2) (**9**) from the bark and leaves of *Syzigium claviflorum*, which showed a significant effect against HIV (EC_50_: 1.4 μM) [17]. Since then, many natural PTs have been reported to have antiviral activities.

**Figure 3:**
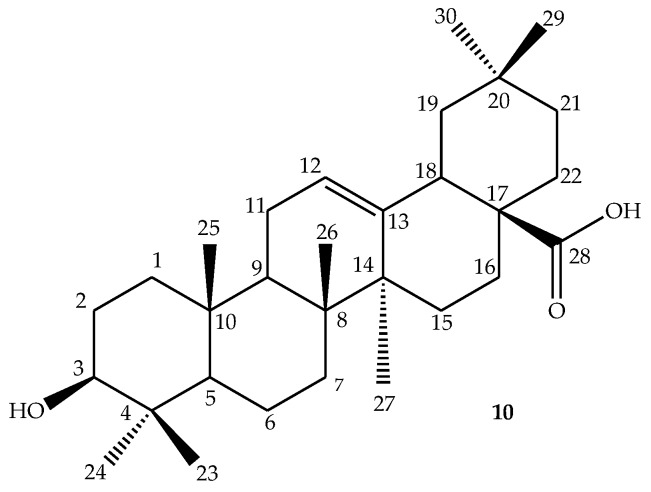
Structure of oleanolic acid (OA).

One natural PT is Oleanolic acid (OA) (Figure 3), which possesses many interesting biological activities, such as antiviral [18], anti-inflammatory, analgesic [18,19], antibacterial [20,21], anti-cancer [22,23,24,25,26], anti-oxidation [27,28], antimicrobial [29,30,31], and cardioprotective activities [32]. Chen et al. reported that OA provides extraordinary protection against acute and chronic liver injury, and can be used as an oral medication for the treatment of human liver disorders [33]. OA is isolated from more than 1600 different plant species [33,34,35,36,37], and is non-toxic and moderately water-soluble [19]. Table 1 below shows some medicinal plants containing OA as an active constituent, and their biological activities. In this study we review modifications of the basic oleanolic acid structure that have been made. 

### Analogues of Oleanolic Acid

**Figure 4:**
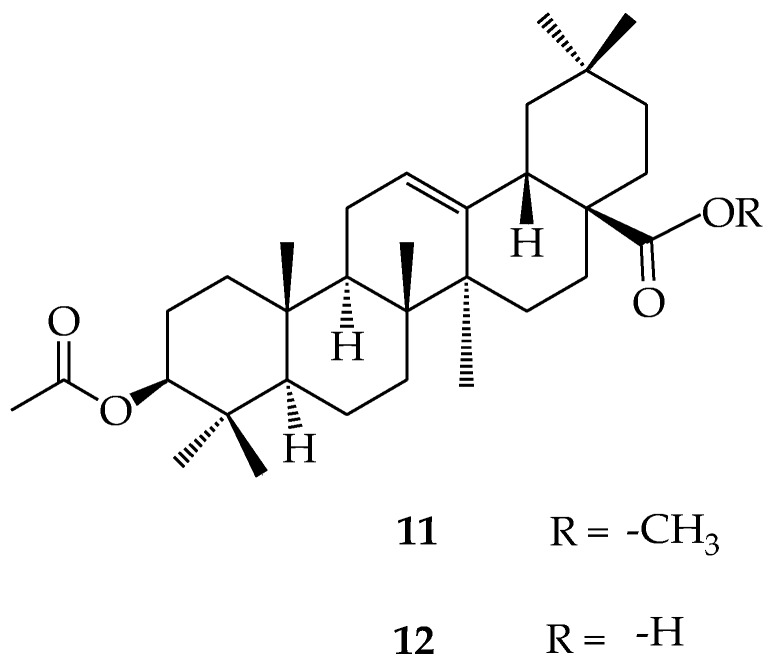
3-acetoxy, 28-methyloleanolic acid (**11**); 3-acetoxyoleanolic acid **(12**).

OA (**10**) has three active sites (i.e., the hydroxyl C-3 in ring **A**, the alkene C12-C13 in ring **C** and carboxylic acid C-28), which can be modified in order to change its physical structure and improve its biological effects [47,48,49]. Many analogues of OA have been synthesized and tested for numerous biological activities [50]. It has been reported in literature that OA is a good precursor molecule for semi-synthetic modifications due to its multiple biological properties, availability, and low production cost [51]. Nkeh-Chungag et al. reported the methylation and acetylation of OA originally isolated from *Syzygium aromaticum* (clove), which resulted in two compounds (Figure 4) (**11**) and (**12**). Both compounds exhibited better in vivo/in vitro anti-inflammatory and membrane-stabilizing properties, respectively, when compared to OA [52].

**Figure 5:**
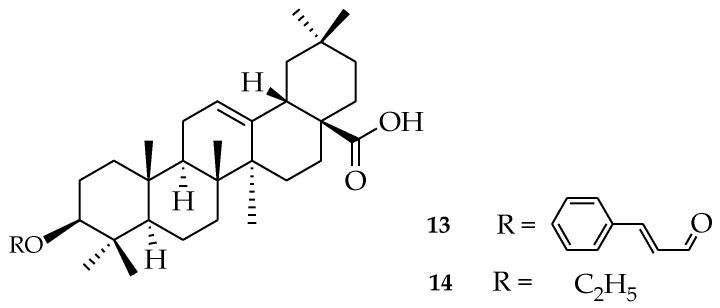
(3b)-3-{[(2*E*)-3-phenylprop-2-enoyl]oxy}olean-12-en-28-oic acid (**13**), (3b)-3-ethoxyolean-12-en-28-oic acid (**14**).

Modification of OA has resulted in compounds with biological activity, such as antidiabetic activity. Yolanda et al. synthesized analogues of OA (three ethers and four esters on hydroxyl C3 in ring **A**, three esters from the carboxyl group C-28, and corresponding primary alcohol), derived from the reduction of carboxylic acid with LiAlH_4_. Cinnamoyl ester (**13**) and ethyl ether (**14**) (Figure 5) were found to be the most PTP-1B inhibitors. The in vitro inhibitory effect of compound **13** was significant, and it substantially lowered blood glucose levels in vivo experiments when compared to OA. Compound **14** exhibited better inhibitory activity and selectivity over protein-tyrosine phosphatase 1B (PTP-1B), with advanced interaction with site B, in accordance with docking studies [53].

**Figure 6:**
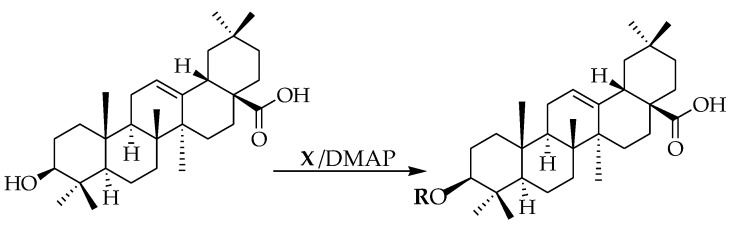
Modification of OA.

The modification of oleanolic acid also resulted in potent antibacterial agents. Hichri et al. explored the effect of introducing an acyl substituent at the hydroxyl C-3 in ring **A** of OA. A sequence of diverse triterpenic acid esters were prepared from oleanolic acid using suitable cyclic anhydrides, acid chlorides, and *N*,*N*-dimethyl-4-aminopyridine (DMAP) as a catalyst (Figure 6 and Table 2) [29]. OA and its acylated analogues were screened for their antimicrobial activity against five fungal plant pathogens, and two Gram-positive and two Gram-negative bacteria. Compound **15** with sulfur and chlorine atom(s), ((3b)-3-((thiophene-2-carbonyl)oxy)-olean-12-en-28-oic acid, was found to be an effective antibacterial agent, and the most active antifungal compound. It exhibited good activity against *A. niger, P. italicum, P. digitatum, A. flavus, and T. harzianum.*

## 2. Phytochemical Studies and Anti-Viral Activities of OA

### 2.1. Anti-HIV Activity

**Figure 7:**
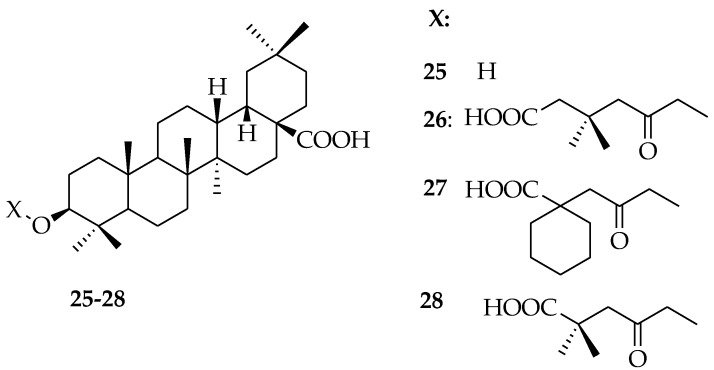
Oleanolic derivatives with anti-HIV activity.

Due to the increase in the occurrence of drug-resistant virus strains, the improvement of effective treatments for the HIV infection is dependent on the identification of novel biomedical agents with low toxicity. Synthesis of oleanolic acid, as well as other closely-related triterpenes, such as betulinic acid and dihydrobetulinic acid, has led to anti-HIV agents [53,54,55]. Zhu et al. synthesized derivatives of OA. These authors modified the C12-C13 double bond of OA yielding compound **25**, which was 3-fold more active than OA. Esterification of **25** with anhydrides resulted in compounds **26**–**28**, which were 5-fold more active than OA with **28** showing remarkable activity [56] (Figure 7). 

**Figure 8:**
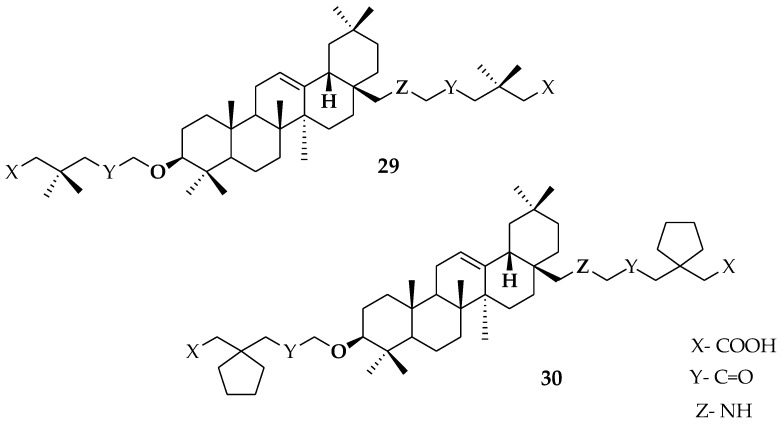
Previously derived OA analogues.

Compound **25** was further modified by converting the C28-carboxyl group to an aminomethyl group, resulting in compounds **29** and **30**, which were greater than 10-fold more active when compared to OA (Figure 8). 

**Figure 9:**
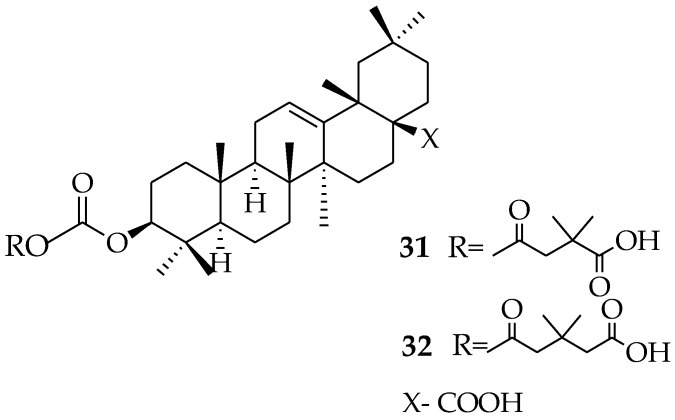
Previously modified anti-HIV triterpene derivatives.

Yu et al. in their structure-activity relationship study of effective anti-HIV agents, synthesized and evaluated new triterpene derivatives in vitro for antiviral activity. OA analogue compound **31** was inactive, while OA derivative **32** exhibited an EC_50_ value of 0.32 μM, indicating that OA is a promising anti-HIV inhibitor [56,57]. These compounds are potential therapeutics that would benefit from further studies in vivo (Figure 9).

Kashiwada et al. prepared several 3-*O*-acyl-ursolic acids and evaluated their anti-HIV activity. The most potent compound indicated an EC_50_ value of 0.31 µM and a TI of 155.5 [58]. In another report by Kashiwada et al., OA derivatives inhibited HIV-1 replication in acutely infected H9 cells. OA-triterpenes isolated from the leaves of *S. claviflorum* exhibited potent anti-HIV activity, which further revealed the potential of OA derivatives for the treatment of HIV [59,60]. 

### 2.2. Anti-Influenza

**Figure 10:**
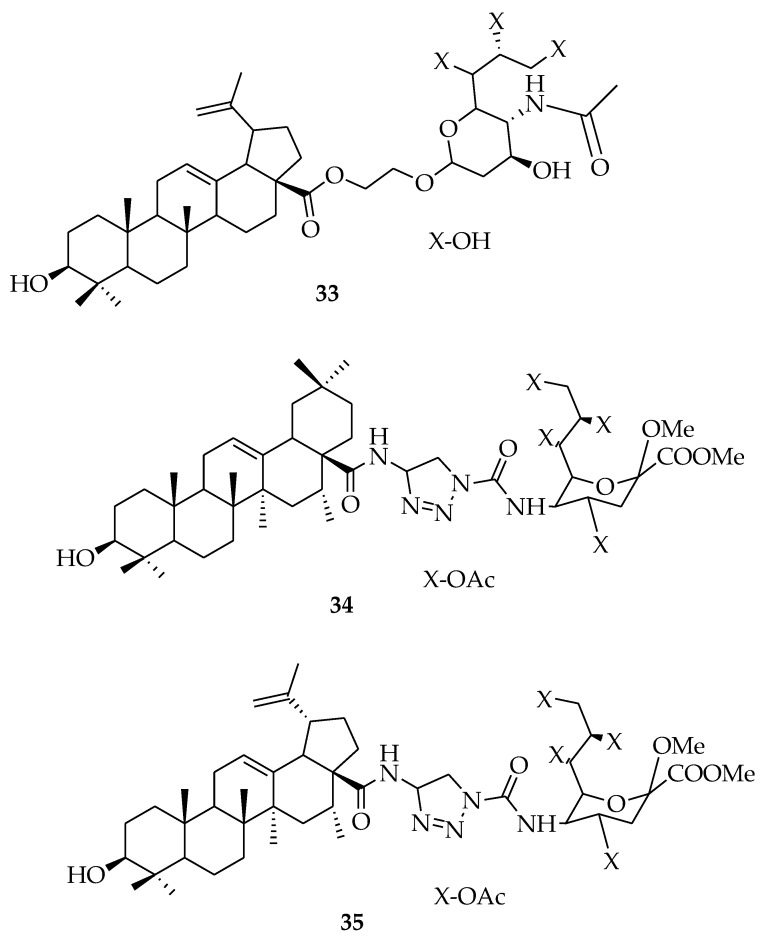
OA derivatives with anti-influenza activity.

The influenza virus is a lethal respiratory virus capable of triggering significant damage to the population. Vaccines and antiviral agents are important for controlling the outbreak of influenza. The research into plant-based drugs against the influenza virus is promising, as some plants have been proven to have anti-influenza properties, some of these include: *Aster spathulifolius*, *Pinus thunbergia, Thuja orientalis* [61], *Allium fistulosum* [62], *Sambucus nigra* [63], and *Psidium guajava* [64]. OA and other molecules, such as chlorogenic acid baicalein and quercetin, are regarded as active constituents in traditional Chinese folk medicine, which have been proven to be effective antiviral agents according to clinical data [65]. These molecules have been found to be potential neuraminidase inhibitors, which could be helpful for anti-influenza medicine development [66]. Yang et al. also reported that OA works as a broad-spectrum entry inhibitor of influenza viruses [67]. Han et al. conjugated sialic acid with OA and its analogues (Figure 9). In vitro evaluation of the compounds on the influenza A/WSN/33 (H1N1) virus in MDCK cell culture revealed compound **33** as the most potent compound, with an IC_50_ of 41.2 μM. It acted as an influenza virus entry inhibitor by inhibiting the binding of the influenza virus hemagglutinin protein to host cells [68]. In a similar report by Han et al., compounds **34** and **35** displayed anti-influenza activity against the A/WSN/33 (H1N1) virus. C-5 acetylamide and C-9 hydroxy of sialic acid were useful for their binding with hemagglutinin during viral entry into host cells [69] (Figure 10). 

### 2.3. Anti-Hepatitis 

Previous reports estimated that approximately 170 million people across the world are infected with the hepatitis C virus (HCV), which causes deaths globally [70]. Since the formation of Ribavirin (RBV) and PEGylated interferon alpha (IFN-α) as the standard drugs for the medical treatment of HCV in 1990, large efforts have been made to find new and more effective drugs. New effective HCV inhibitors belonging to direct-acting antivirals (DAAs), which include Boceprevir, Ledipasvir, Sofosbuvir, Telaprevir, Simeprevir, and Daclatasvir, have been developed in recent years [71]. Although great progress in anti-HCV remedies has been surely attained, many barriers nevertheless exist. Monotherapy with DAAs is linked with the rapid occurrence of drug-resistant viral mutations, thus the medical utility of DAAs is usually restricted to mixture regimens, which is associated with extra side effects, drug-drug interactions, high costs, and availability. Therefore, new therapeutic techniques consisting of various HCV inhibitors focused on particular stages of the HCV life cycle, with more effectiveness and wider availability, are still in demand to overcome those obstacles. Historically, many active modern drugs have been established from compounds initially isolated from plants, and now there is still more interest in finding new drugs from herbal sources for the treatment of various human diseases. Many natural compounds have been identified with antiviral effects globally, which includes anti-HCV activity. OA was an important compound in many traditional Chinese hepatic protective drugs, and was considered to play a therapeutic role in many diseases. Indeed, *Plantago major* L., a well-known traditional Chinese remedy, has been used for the treatment of many diseases including the cold and viral hepatitis [72]. OA and its isomer, ursolic acid, were identified as active compounds of *Fructus Ligustri Lucidi* (FLL), and were indicated as two antiviral compounds that seriously suppressed the duplication of the Hepatitis C Virus (HCV) genotype 1b replicon, and the HCV genotype 2a JFH1 virus. Furthermore, these compounds exhibited anti-HCV properties, partly by suppressing HCV NS5B RdRp properties, as noncompetitive inhibitors. Therefore, their results confirmed that natural products of OA and ursolic acid are potential HCV antivirals [18].

**Figure 11:**
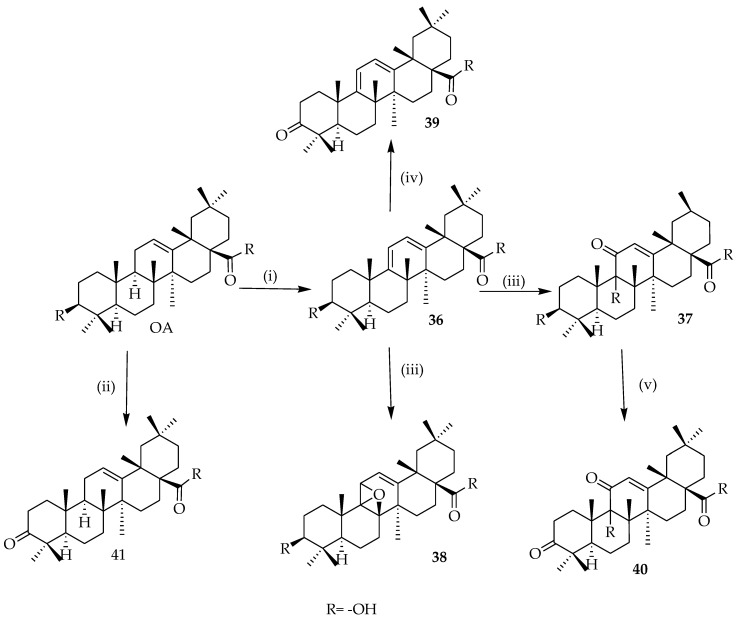
Synthesis route to derivatives. Reagents and conditions: (i) *N*-Bromosuccinimide (NBS), CCl_4_, light, reflux, 4 h; (ii) chromic acid solution, acetone, 0 °C, 1 h; (iii) Eosin Y, dichloromethane, light, 10 h; (iv) chromic acid solution, acetone, 0 °C, 1 h; (v) chromic acid solution, acetone, 0 °C, 1 h.

Yan et al. (2006) reported interesting work on the synthesis of OA structures (Figure 11). These researchers proposed a series of OA analogues which were synthesized by various reactions. All tested analogues (**38**–**41**) inhibited the secretion of HBsAg, and also decreased the secretion of HBeAg [73]. Compound **39** showed major inhibition of HBV DNA duplication, which was significant when compared to the reference drug. Due to its great performance in vivo and in vitro, compound **39** is a potential novel anti-Hepatitis B virus drug candidate, with a different mechanism of action, which needs further investigation. 

### 2.4. Anti-Herpes

**Figure 12:**
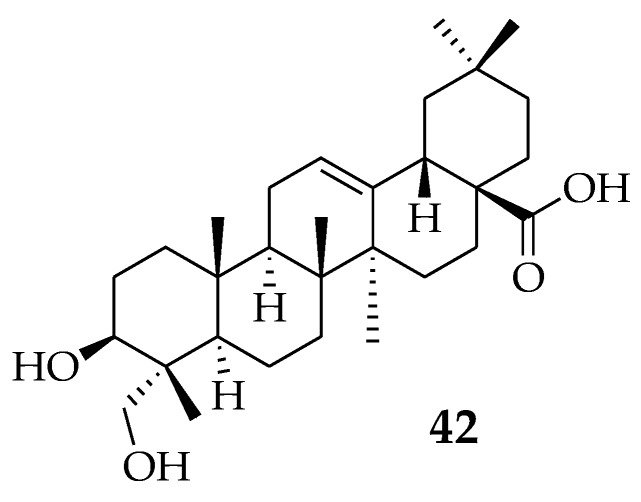
Hederagenin.

Herpes simplex virus (HSV) has two serotypes (HSV-1 and HSV-2), which target the oral and genital mucous membranes of humans, create latent infections in the sensory neurons, and may reactivate to cause recurring infections at the primary site [74]. The effects of genital herpes as a human health threat are increasing because of its interrelationship with HIV. HSV has been well treated with acyclovir since 1970 [75]. Some licensed anti-herpes virus drugs, such as ganciclovir, cidofovir, and foscarnet, that destroy herpes virus DNA polymerases, also have toxicity in long-term usage [76]. Therefore, novel antiviral drugs from natural sources that have different mechanisms of action are in great demand. Mukherjee et al. isolated OA from methanol (MeOH) extract taken from *Achyranthes aspera* roots. The MeOH extract exhibited a weak anti-herpes virus effect (EC_50_ 64.4 g/mL for Herpes simplex virus 1 (HSV-1) and 72.8 g/mL for Herpes simplex virus 2 (HSV-2)), whereas OA showed potent anti-herpes virus activity against both HSV-1 (EC_50_ 6.8 g/mL) and HSV-2 (EC_50_ 7.8 g/mL) [74]. Keda et al., also examined 15 oleanane-type triterpenoids, and their derivatives, for anti-herpes simplex virus type 1 (HSV-1) activities. OA and its derivative Hederagenin (Figure 12) (**42**) were found to exhibit moderate anti-HSV-1 activity [77].

## 3. Conclusions

Triterpenoids are an important group of compounds widely found in nature. Previous reports on triterpenoids showed that OA and its analogues have countless beneficial effects, such as antiviral, anti-inflammatory, antitumor promotion, anticancer, and so forth. OA is relatively non-toxic and has been sold in China as a therapeutic for the treatment of human hepatitis. There are few reports on the antiviral activities of OA and its analogues. The treatment of viral diseases continues to present problems in current medicine, with various studies showing a great increase in the occurrence of drug resistant viruses [78]. The modification of the hydroxyl and carboxylic acid functional groups on OA has resulted in potent compounds with antiviral activity. The anti-HIV activity of the synthesized compounds is via the inhibition of HIV-1 replication. The compounds synthesized also hindered virus entry by inhibiting the binding of influenza virus hemagglutinin protein to the host cells. However, a thorough antiviral mode of action of these compounds needs further investigation. The initial information obtained from in vivo and in vitro studies of OA derivatives is promising. Therefore, further studies are needed into OA, and its analogues from natural products, as an antiviral agent for various human diseases.

## Figures and Tables

**Table 1:** Several plants where OA was reported, the plant parts used, and their biological activities.

Plant Species (Family)	Biological Activity	Plant Parts Used	References
*Oleaeuropaea* L. (Oleaceae)	Anticancer, antimicrobial, anti-diabetic	Fruits and leaves	[22,28,37,38]
*Fabiana imbricata* R. et P. (Solanaceae)	Antiviral, antitumor, and antihyperlipidemic	Leaves and flowers	[39]
*Syzygium aromaticum* (Myrtaceae)	Antinociceptive, Anti-inflammatory, antihypertensive, and antioxidant	Flower buds and leaves	[18,39,40]
*Ligustrum lucidum Ait* (Oleaceae)	Anti-inflammatory, antioxidative, antiprotozoal, antimutagenic, and anticancer	Fruits and leaves	[41]
*Viscum album* (Santalaceae)	Anti-tumor, analgesic, and anti-inflammatory	Leaves and stems	[19,41,42]
*Phyllanthus amarus* (Phyllanthaceae*)*	Anti-diabetes	Leaves or aerial	[43]
*Punica granatum* L. (Punicaceae)	Antioxidant activity	Fruit	[27]
*Rosmarinus officinalis* L. (Lamiaceae)	Anti-inflammatory, hepatoprotective, gastroprotective, antiulcer	Leaves, flowers, stems, branches.	[28]
*Gentiana lutea (*Gentianaceae)	Antimicrobial	Dried root and rhizome	[30]
*L. camara* (Verbenaceae)	Anti-inflammatory, antioxidative, antiprotozoal	Leaves and flowers	[41]
*Viburnum chingii* (Asteraceae)	Antimicrobial	Leaves	[44]
*Siphonodon celastrineus* (Celastraceae)	Anti-inflammatory	Root bark, stem	[45,46]
*Rosa laevigata* (Rosaceae)	Anti-inflammatory	Leaves	[47]
*Fructus Ligustri Lucidi* (FLL)	Anti-hepatitis	Leaves	[18]

**Table 2:** Synthesis of oleanolic acid.

Compound	R	X	Yield (%) ^1^
**15**	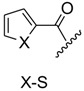	RCl	98
**16**	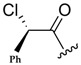	RCl	94
**17**	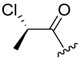	RCl	91
**18**	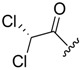	RCl	95
**19**	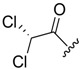	RCl	94
**20**	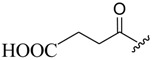	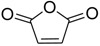	92
**21**	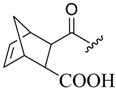	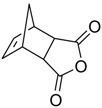	82
**22**	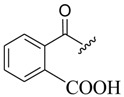	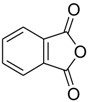	91
**23**	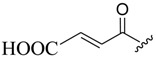	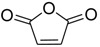	85
**24**	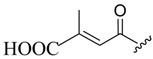	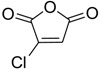	81

**^1^** Obtained percentage yield of product.

## References

[1] Rumlová M., Ruml T. (2018). In vitro methods for testing antiviral drugs. Biotechnol. Adv..

[2] Nováková L., Pavlík J., Chrenková L., Martinec O., Červený L. (2018). Current antiviral drugs and their analysis in biological materials—Part I: Antivirals against respiratory and herpes viruses. J. Pharm. Biomed. Anal..

[3] National Health Laboratory Service. http://www.nhls.ac.za/?page=alerts&id=5&archive=2016&rows=5&pager=5.

[4] Kitazato K., Wang Y., Kobayashi N. (2007). Viral infectious disease and natural products with antiviral activity. Drug Discov. Ther..

[5] Ganjhu R.K., Mudgal P.P., Maity H., Dowarha D., Devadiga S., Nag S., Arunkumar G. (2015). Herbal plants and plant preparations as remedial approach for viral diseases. VirusDisease.

[6] Ahmed-Belkacem A., Ahnou N., Barbotte L., Wychowski C., Pallier C., Brillet R., Pohl R., Pawlotsky J. (2010). Silibinin and Related Compounds Are Direct Inhibitors of Hepatitis C. Gastroenterology.

[7] Morishima C., Shuhart M.C., Wang C.C., Paschal D.M., Apodaca M.C. (2010). Silymarin Inhibits In Vitro T-Cell Proliferation and Cytokine Production in Hepatitis C Virus Infection. Gastroenterology.

[8] Dinh T., Moons N., Kim Y., Borggraeve W.D., Mashentseva A., Andrei G., Snoeck R., Balzarini J., Dehaen W. (2014). Synthesis of triterpenoid triazine derivatives from allobetulone and betulonic acid with biological activities. Bioorg. Med. Chem..

[9] Ruzicka L. (1953). The isoprene rule and the biogenesis of terpenic compounds. Experientia.

[10] Xiao S., Tian Z., Wang Y., Si L., Zhang L., Zhou D. (2018). Recent progress in the antiviral activity and mechanism study of pentacyclic triterpenoids and their derivatives. Med. Res. Rev..

[11] Bishayee A., Ahmed S., Brankov N., Perloff M. (2011). Triterpenoids as potential agents for the chemoprevention and therapy of breast cancer. Front. Biosci..

[12] Xu R., Fazio G.C., Matsuda S.P.T. (2004). On the origins of triterpenoid skeletal diversity. Phytochemistry.

[13] Villa T.G., Feijoo-Siota L., Rama J.L.R., Ageitos J.M. (2017). Antivirals against animal viruses. Biochem. Pharmacol..

[14] Lin L.T., Hsu W.C., Lin C.C. (2014). Antiviral Natural Products and Herbal Medicines. J. Tradit. Complement. Med..

[15] Pompei R., Flore O., Marccialis A.M., Pani A., Loddo B. (1979). Glycyrrhizic acid inhibits virus growth and inactivates virus particles. Nature.

[16] Chen K., Qian S., Yoshiki K., Zhang D.-C., Hu C.-Q., Jin J.-Q., Nozaki H., Kilkuskie R.E., Tramontano E., Cheng Y.-C. (1992). Anti-aids agents, 6. salaspermic acid, an anti-HIV principle from tripterygium wilfordii, and the structure-activity correlation with its related compounds. Prod. J. Nat..

[17] Fujioka T., Kashiwada Y., Robert E.K., Cosentino L.M., Ballas L.M., Jiang J.B., Lanzen W.P., Cheen I., Lee K. (1994). Anti- aids agents, 11 betulinic acid and plantanic acid as anti-HIV principles from syzygium claviflorum, and the anti-HIV activity of structurally relate triterpenoids. J. Nat. Prod..

[18] Kong L., Liao Q., Zhang Y., Sun R., Zhu X., Zhang Q., Wang J., Wu X., Fang X., Zhu Y. (2013). Oleanolic acid and ursolic acid: Novel hepatitis C virus antivirals that inhibit NS5B activity. Antiviral Res..

[19] Rali S., Oyedeji O.O., Aremu O.O., Oyedeji A.O., Nkeh-Chungag B.N. (2016). Semisynthesis of derivatives of oleanolic acid from Syzygium aromaticum and their antinociceptive and anti-inflammatory properties. Mediators Inflamm..

[20] Kim S., Lee H., Lee S., Yoon Y., Choi K.H. (2015). Antimicrobial action of oleanolic acid on *Listeria monocytogenes*, *Enterococcus faecium*, and *Enterococcus faecalis*. PLoS ONE.

[21] Hichri F., Ben H., Cheriaa J., Jegham S., Mighri Z. (2003). Antibacterial activities of a few prepared derivatives of oleanolic acid and of other natural triterpenic compounds. C. R. Chim..

[22] Li X., Song Y., Zhang P., Zhu H., Chen L., Xiao Y., Xing Y. (2016). Oleanolic acid inhibits cell survival and proliferation of prostate cancer cells in vitro and in vivo through the PI3K/Akt pathway. Tumor Biol..

[23] Chouaïb K., Romdhane A., Dlelmasure S., Dutartre P., Elie N., Toutboul D., Ben H., Ali M. (2016). Regiospecific synthesis, anti-inflammatory and anticancer evaluation of novel 3,5-disubstituted isoxazoles from the natural maslinic and oleanolic acids. Ind. Crops Prod..

[24] Guo Y., Han B., Luo K., Ren Z., Cai L., Sun L. (2017). NOX2-ROS-HIF-1α signaling is critical for the inhibitory effect of oleanolic acid on rectal cancer cell proliferation. Biomed. Pharmacother..

[25] Shanmugam M.K., Dai X., Kumar A.P., Tan B. K.H., Sethi G., Bishayee A. (2014). Oleanolic acid and its synthetic derivatives for the prevention and therapy of cancer: Preclinical and clinical evidence. Cancer Lett..

[26] Oprean C., Mioc M., Csányi E., Ambrus R., Bojin F., Tatu C., Critea M., Ivan A., Dancui C., Dehelean C. (2015). Improvement of ursolic and oleanolic acids′ antitumor activity by complexation with hydrophilic cyclodextrins. Biomed. Pharmacother..

[27] Pattnaik B., Nayak L.V., Sistla R., Mallavadhani V.U. (2016). Bioorganic Chemistry Synthesis of ring-C modified oleanolic acid derivatives and their cytotoxic evaluation. Bioorg. Chem..

[28] Fu Q., Zhang L., Cheng N., Jia M., Zhang Y. (2014). Extraction optimization of oleanolic and ursolic acids from pomegranate (*Punica granatum* L.) flowers. Food Bioprod. Process..

[29] Bernatoniene J., Cizauskaite U., Ivanauskas L., Jakstas V., Kalveniene Z., Kopustinskiene D.M. (2016). Novel approaches to optimize extraction processes of ursolic, oleanolic and rosmarinic acids from Rosmarinus officinalis leaves. Ind. Crops Prod..

[30] Chouab K., Hichri F., Nguir A., Daami-Remadi M., Elie N., Touboul D., Ben J.H., Hamza M. (2015). Semi-synthesis of new antimicrobial esters from the natural oleanolic and maslinic acids. Food Chem..

[31] Weckesser S., Engel K., Simon-haarhaus B., Wittmer A., Pelz K., Schempp C.M. (2007). Screening of plant extracts for antimicrobial activity against bacteria and yeasts with dermatological relevance. Phytomedicine.

[32] Aisha A.F.A., Abu-salah K.M., Salman A. (2012). Syzygium aromaticum extracts as good source of betulinic acid and potential anti-breast cancer. Rev. Bras. Farmacogn..

[33] Chen P., Zeng H., Wang Y., Fan X., Xu C., Deng R., Zhou X., Bi H., Huang M. (2014). Low Dose of Oleanolic Acid Protects against Lithocholic Acid–Induced Cholestasis in Mice: Potential Involvement of Nuclear Factor-E2-Related Factor 2-Mediated Upregulation of Multidrug Resistance-Associated Proteins. Am. Soc. Pharmacol. Exp. Ther..

[34] Sheng H., Sun H. (2011). Synthesis, biology and clinical significance of pentacyclic triterpenes: A multi-target approach to prevention and treatment of metabolic and vascular diseases. Nat. Prod. Rep..

[35] Liu J. (2005). Oleanolic acid and ursolic acid: Research perspectives. J. Ethnopharmacol..

[36] Fukushima E.O., Seki H., Ohyama K., Ono E., Umemoto N., Mizutani M., Saito K., Muranaka T. (2011). CYP716A Subfamily Members are Multifunctional Oxidases in Triterpenoid Biosynthesis. Plant Cell Physiol..

[37] Jesus J.A., Lago J.H.G., Laurenti M.D., Yamamoto E.S., Passero L.F.D. (2015). Antimicrobial activity of oleanolic and ursolic acids: An update. Evid.-Based Complement. Altern. Med..

[38] Strehle A., Thomas C., Sato H., Lobstein A., Wagner A., Mioskowski C., Auwerx J. (2007). Anti-hyperglycemic activity of a TGR5 agonist isolated from *Olea europaea*. Biochem. Biophys. Res. Commun..

[39] Sánchez M., Theoduloz C., Schmeda-hirschmann G., Razmilic I., Yáñez T., Rodríguez J.A. (2006). Gastroprotective and ulcer-healing activity of oleanolic acid derivatives: In vitro–in vivo relationships. Life Sci..

[40] Somova L.O., Nadar A., Rammanan P., Shode F.O. (2003). Cardiovascular, antihyperlipidemic and antioxidant effects of oleanolic and ursolic acids in experimental. Phytomedicine.

[41] Banik R.M., Pandey D.K. (2008). Optimizing conditions for oleanolic acid extraction from *Lantana camara* roots using response surface methodology. Ind. Crops Prod..

[42] Jäger S., Winkler K. (2007). Solubility Studies of Oleanolic Acid and Betulinic Acid in Aqueous Solutions and Plant Extracts of *Viscum album* L.. Planta Med..

[43] Ali H., Houghton P.J., Soumyanath A. (2006). Amylase inhibitory activity of some Malaysian plants used to treat diabetes with particular reference to *Phyllanthus amarus*. J. Ethnopharmacol..

[44] Chen X.Q., Li Y., He J., Cheng X., Wang K., Li M.M., Pan Z.H., Peng L.Y., Zhao Q.S. (2011). Triterpenoids and diterpenoids from *Viburnum chingii*. Chem. Pharm. Bull..

[45] Niampoka C., Suttisri R., Bavovada R., Takayama H., Aimi N. (2005). Potentially cytotoxic triterpenoids from the root bark of *Siphonodon celastrineus* Griff. Arch. Pharm. Res..

[46] Kaweetripob W., Mahidol C., Prawat H., Ruchirawat S. (2013). Lupane, friedelane, oleanane, and ursane triterpenes from the stem of *Siphonodon celastrineus* Griff. Phytochemistry.

[47] Yan M., Zhu Y., Zhang H.J., Jiao W.H., Han B.N., Liu Z.X., Qiu F., Chen W.S., Lin H.W. (2013). Anti-inflammatory secondary metabolites from the leaves of *Rosa laevigata*. Bioorg. Med. Chem..

[48] Liby K.T., Sporn M.B. (2012). Synthetic Oleanane Triterpenoids: Multifunctional Drugs with a Broad Range of Applications for Prevention and Treatment of Chronic Disease. Pharmacol. Rev..

[49] Suh N., Wng Y., Honda T., Gribble G.W. (1999). Advances in Brief A Novel Synthetic Oleanane Triterpenoid, 2-Cyano-3,12-dioxoolean-1,9-dien-28-oic Acid, with Potent Differentiating, Antiproliferative, and Anti-Inflammatory Activity. Cancer Res..

[50] Chen J., Liu J., Zhang L., Wu G., Hua W. (2006). Pentacyclic triterpenes. Part 3: Synthesis and biological evaluation of oleanolic acid derivatives as novel inhibitors of glycogen phosphorylase. Bioorg. Med. Chem. Lett..

[51] Pollier J., Goossens A. (2012). Phytochemistry Oleanolic acid. Phytochemistry.

[52] Nkeh-chungag B.N., Oyedeji O.O., Oyedeji A.O., Ndebia E.J. (2015). Anti-Inflammatory and Membrane-Stabilizing Properties of Two Semisynthetic Derivatives of Oleanolic Acid. Inflammation.

[53] Zhao H., Holmes S.S., Baker G.A., Challa S., Bose H.S., Song Z. (2012). Ionic derivatives of betulinic acid as novel HIV-1 protease inhibitors. J. Enzyme Inhib. Med. Chem..

[54] Aiken C., Chen C.H. (2005). Betulinic acid derivatives as HIV-1 antivirals. Trends Mol. Med..

[55] Yogeeswari P., Sriram D. (2005). Betulinic Acid and Its Derivatives: A Review on their Biological Properties. Curr. Med. Chem..

[56] Zhu Y., Shen J., Wang H., Mark L., Lee K. (2001). Synthesis and Anti-HIV Activity of Oleanolic Acid Derivatives. Bioorg. Med. Chem. Lett..

[57] Yu D., Sakurai Y., Chen C., Chang F., Huang L., Kashiwad Y., Kuo-Hsiung L. (2006). Anti-AIDS Agents 69. Moronic Acid and Other Triterpene Derivatives as Novel Potent Anti-HIV Agents. J. Med. Chem..

[58] Kashiwada Y., Nagao T., Hashimoto A., Ikeshiro Y., Okabe H., Cosentino L.M., Lee K.H. (2000). Anti-AIDS agents 38. Anti-HIV activity of 3-*O*-acyl ursolic acid derivatives. J. Nat. Prod..

[59] Kashiwada Y., Wang H.K., Nagao T., Kitanaka S., Yasuda I., Fujioka T., Yamagishi T., Cosentino L.M., Kozuka M., Okabe H. (1998). Anti-HIV activity of oleanolic acid, pomolic acid, and structurally related triterpenoids. J. Nat. Prod..

[60] Sultana N., Ata A. (2008). Oleanolic acid and related derivatives as medicinally important compounds. J. Enzyme Inhib. Med. Chem..

[61] Lee S., Song D., Poo H. (2013). Antiviral Activity of the Plant Extracts from *Thuja orientalis*, *Aster spathulifolius*, and *Pinus thunbergii* against Influenza Virus A/PR/8/34. J. Microbiol. Biotechnol..

[62] Lee J., Miyake S., Umetsu R., Hayashi K., Chijimatsu T. (2012). Anti-influenza A virus effects of fructan from Welsh onion (*Allium fistulosum* L.). Food Chem..

[63] Kinoshita E., Hayashi K., Katayama H., Hayashi T., Obata A. (2012). Anti-Influenza Virus Effects of Elderberry Juice and Its Fractions. Biosci. Biotechnol. Biochem..

[64] Sriwilaijaroen N., Fukumoto S., Kumagai K., Hiramatsu H., Odagiri T. (2012). Antiviral effects of *Psidium guajava* Linn. (guava) tea on the growth of clinical isolated H1N1 viruses: Its role in viral hemagglutination and neuraminidase inhibition. Antiviral Res..

[65] Wang X., Jia W., Zhao A., Wang X. (2006). Anti-influenza agents from plants and traditional Chinese medicine. Phyther. Res..

[66] Liu Z., Zhao J., Li W., Wang X., Xu J., Xie J., Tao K., Shen L., Zhang R. (2015). Molecular docking of potential inhibitors for influenza H7N9. Comput. Math. Methods Med..

[67] Yang Y., He H., Chang H., Yu Y., Yang M., He Y. (2018). Multivalent oleanolic acid human serum albumin conjugate as nonglycosylated neomucin for influenza virus capture and entry inhibition. Eur. J. Med. Chem..

[68] Han X., Shi Y., Si L., Fan Z., Wang H., Xu R., Jiao P., Meng K., Tian Z., Zhou X. (2016). Design, synthesis and biological activity evaluation of novel conjugated sialic acid and pentacyclic triterpene derivatives as anti-influenza entry inhibitors. MedChemComm.

[69] Han X., Si L.L., Shi Y.Y., Fan Z.B., Wang S.X., Tian Z.Y., Li M., Sun J.Q., Jiao P.X., Ran F.X. (2017). Synthesis and in vitro anti-influenza virus evaluation of novel sialic acid (C-5 and C-9)-pentacyclic triterpene derivatives. Molecules.

[70] Hattori M., Ma C., Wei Y., Salah R., Dine E., Sato N. (2013). Survey of Anti-HIV and Anti-HCV Compounds from Natural Sources. Can. Chem. Trans..

[71] Pastuch-Gawolek G., Chaubey B., Szewczyk B., Krol E. (2017). Novel thioglycosyl analogs of glycosyltransferase substrates as antiviral compounds against classical swine fever virus and hepatitis C virus. Eur. J. Med. Chem..

[72] Chiang L.C., Chiang W., Chang M.Y., Ng L.T., Lin C.C. (2002). Antiviral activity of Plantago major extracts and related compounds in vitro. Antiviral Res..

[73] Yan W., Zhang C., Li B., Xu X., Liang M., Gu S., Chu S., Xu B., Ren J., Wang P. (2016). A Series of Oleanolic Acid Derivatives as Anti-Hepatitis B Virus Agents: Design, Synthesis, and in Vitro and in Vivo Biological Evaluation. Molecules.

[74] Mukherjee H., Ojha D., Bag P., Chandel H.S., Bhattacharyya S., Chatterjee T.K., Mukherjee P.K., Chakraborti S., Chattopadhyay D. (2013). Anti-herpes virus activities of *Achyranthes aspera*: An Indian ethnomedicine, and its triterpene acid. Microbiol. Res..

[75] Whitley R.J., Roizman B. (2001). Herpes simplex virus infections. Lancet.

[76] De Clercq E. (2004). Antiviral drugs in current clinical use. J. Clin. Virol..

[77] Keda T.I., Okomizo K.Y., Suchihashi M.O., Injo J.K. (2005). Anti-herpes Virus Type 1 Activity of Oleanane-Type Triterpenoids. Biol. Pharm. Bull..

[78] Sarrazin C. (2016). The importance of resistance to direct antiviral drugs in HCV infection in clinical practice. J. Hepatol..

